# Baseline Serum Osteopontin Levels Predict the Clinical Effectiveness of Tocilizumab but Not Infliximab in Biologic-Naïve Patients with Rheumatoid Arthritis: A Single-Center Prospective Study at 1 Year (the Keio First-Bio Cohort Study)

**DOI:** 10.1371/journal.pone.0145468

**Published:** 2015-12-23

**Authors:** Keisuke Izumi, Yuko Kaneko, Misato Hashizume, Keiko Yoshimoto, Tsutomu Takeuchi

**Affiliations:** 1 Division of Rheumatology, Department of Internal Medicine, Keio University School of Medicine, Tokyo, Japan; 2 Department of Connective Tissue Diseases, National Tokyo Medical Center, Tokyo, Japan; 3 Product Research Department, Fuji-Gotemba Research Laboratories, Chugai Pharmaceutical Co., Ltd., Gotemba, Japan; Nippon Medical School Graduate School of Medicine, JAPAN

## Abstract

**Objective:**

To explore the baseline predictors of clinical effectiveness after tocilizumab or infliximab treatment in biologic-naïve rheumatoid arthritis patients.

**Methods:**

Consecutive biologic-naïve patients with rheumatoid arthritis initiating infliximab (n = 57) or tocilizumab (n = 70) treatment were included in our prospective cohort study. Our cohort started in February 2010, and the patients observed for at least 1 year as of April 2013 were analysed. We assessed baseline variables including patients' characteristics (age, sex, disease duration, prednisolone dose, methotrexate dose, other disease-modifying antirheumatic drug use, Clinical Disease Activity Index [CDAI]) and serum biomarker levels (C-reactive protein, immunoglobulin M-rheumatoid factor, anti-cyclic citrullinated protein/peptide antibodies, interferon-γ, interleukin (IL)-1β, IL-2, IL-6, IL-8, IL-10, IL-17, tumor necrosis factor-α, soluble intercellular adhesion molecule-1, bone alkaline phosphatase, osteonectin, osteopontin) to extract factors associated with clinical remission (CDAI≤2.8) at 1 year using univariate analyses, and the extracted factors were entered into a multivariate logistic regression model. Similar analyses were also performed for Simplified Disease Activity Index (SDAI) remission (≤3.3) and Disease Activity Score with 28 joint counts, erythrocyte sedimentation rate (DAS28-ESR) remission (<2.6).

**Results:**

There were no significant differences in the baseline characteristics except for methotrexate use between the groups. In the multivariate analyses, the low baseline osteopontin levels (OR 0.9145, 95% CI 0.8399–0.9857) were identified as predictors of CDAI remission in the tocilizumab group, whereas no predictors of CDAI remission were found in the infliximab group. Similar results were obtained when using SDAI and DAS28-ESR remission criteria.

**Conclusion:**

Baseline low serum osteopontin levels predict clinical remission 1 year after tocilizumab treatment and not infliximab treatment in biologic-naïve patients with rheumatoid arthritis. Our prediction model provided insights into how to optimize the choice of biologics and warrants external validation in other cohorts.

## Introduction

Rheumatoid arthritis (RA) is an autoimmune disorder of the synovium that is characterized by the proliferation of synoviocytes and by the infiltration of inflammatory cells into the joint[1]. Various cytokines have been reported to play an important role in the regulation of inflammatory cells[2]. Biologics targeting tumor necrosis factor (TNF) and interleukin (IL)-6 have made considerable progress in the treatment of RA. However, responses to each biologic agent vary by individual. Therefore, making an optimal choice of biologics has been expected to capture a therapeutic window of opportunity and to lead to cost-effective medical care.

Hence, we aimed to identify useful baseline variables and biomarkers measurable in the peripheral blood samples to predict clinical remissions in biologic-naïve RA patients after treatment with tocilizumab (TCZ) or infliximab (IFX).

## Materials and Methods

### Patients

Our study protocol was approved by the ethics committee at Keio University School of Medicine. Written informed consent was obtained from each of the patient. The 2008 Declaration of Helsinki and the 2008 Ethical Guidelines for Clinical Research by the Japanese Ministry of Health, Labour and Welfare were followed. Consecutive RA patients who fulfilled the 1987[3] or 2010 American College of Rheumatology/European League Against Rheumatism classification criteria[4], and who initiated IFX or TCZ as the first biologic agent were enrolled in the cohort. IFX was infused at a dose of 3 mg/kg at weeks 0, 2, and 6 and subsequently every 8 weeks with methotrexate (MTX), and TCZ was infused at a dose of 8 mg/kg at every 4 weeks. Choice of biologics was at physicians’ discretion. Our cohort was initiated in February 2010, and the patients who had been observed for at least 1 year as of April 2013 were analyzed.

### Serum Assays

Serum samples were collected immediately before the infusions at baseline. After collection, all of the samples were stored immediately at -30°C in our laboratory. Serum biomarkers (interferon-γ, IL-1β, IL-2, IL-6, IL-8, IL-10, IL-17, TNF-α, soluble intercellular adhesion molecule-1, osteonectin, full-length osteopontin [OPN], and bone alkaline phosphatase) were measured by an electrochemiluminescence assay with an Ultra-Sensitive Kit^®^ (Meso Scale Discovery, MD, USA). In this assay, an immunoglobulin inhibiting reagent (Bioreclamation, NY, USA) was used to block heterophilic antibody interference[5]. C-reactive protein (CRP) was measured by the latex turbidimetric immunoassay, immunoglobulin M-rheumatoid factor (RF; normal range, ≤15 IU/mL) was measured by immunonephelometry, and anti-cyclic citrullinated protein/peptide antibodies (ACPA; normal range <4.5 U/mL) were measured by a chemiluminescent enzyme immunoassay.

### Statistical Analysis

The statistical analyses were performed with JMP V.11.0.0 (SAS Institute Inc., NC, USA). Clinical remission (Clinical Disease Activity Index [CDAI] ≤2.8; Simplified Disease Activity Index [SDAI] ≤3.3; disease activity score with 28 joint counts, erythrocyte sedimentation rate [DAS28-ESR] <2.6) at 1 year was analyzed using the last observation carried forward (LOCF) approach. Kaplan-Meier analysis was used to estimate the retention rates during the first 1 year, and the difference in the retention curves between biologics was examined by the log-rank test. All of the reported P values are two-sided and are considered to be statistically significant for two-sided P<0.05.

A univariate logistic regression analysis was used to extract the potential predictors of clinical remissions, and the extracted predictive factors with P values less than 0.1 in univariate analyses were entered into a multivariate model. A receiver operating characteristics (ROC) curve was developed based on the logistic regression analysis, and the significant cut-off value was determined from the curve.

## Results

### Baseline Characteristics of the Patients

Fifty-seven patients were enrolled in the IFX group, and 70 were in the TCZ group. All of the patients were Japanese. The mean age and disease duration were 55.8±13.5 years and 7.7±8.8 years, respectively, in the IFX group and 56.6±12.6 years and 5.9±6.7 years, respectively, in the TCZ group. There were no differences in the baseline characteristics other than MTX use between the groups; the proportion of MTX use was 100% in the IFX group and 75.7% in the TCZ group (Table 1). In the TCZ group, however, there were no significant differences in the baseline characteristics of the patients with or without MTX. In addition, there were no significant differences in the baseline characteristics between the IFX group and the patients in the TCZ group who received concomitant MTX (S4 Table).

**Table 1:** Overall baseline characteristics of the patients.

	IFX (n = 57)		TCZ (n = 70)		P value
	Mean±SD	Median [IQR]	Mean±SD	Median [IQR]	
Age, years	55.8±13.5	56.0 [47.0–67.0]	56.6±12.6	59.0 [48.8–64.5]	0.81
Women, n (%)	47 (82.5) §		61 (87.1) §		0.47
Duration, years	7.7±8.8	3.1 [0.5–13.6]	5.9±6.7	3.9 [1.5–7.9]	0.98
PSL use, n (%)	11 (19.3) §		22 (31.4) §		0.16
PSL dose in PSL-use patients, mg/day	1.7±4.7	0 [0–0]	1.8±3.4	0 [0–3]	0.24
MTX use, n (%)	57 (100) §		53 (75.7) §		<0.0001*
MTX dose in MTX-use patients, mg/week	8.5±2.1	8 [8–9]	8.5±2.1	8 [8–10]	0.71
Other DMARDs use, n (%)	6 (10.5) §		6 (8.6) §		0.77
SJC (28 joints)	8.3±6.2	7 [4–13]	6.2±4.3	5 [3–8]	0.10
TJC (28 joints)	6.8±6.9	5 [1–11]	5.7±4.2	5 [3–7]	0.83
PhGA (0–10), cm	5.0±2.3	5.0 [3.0–6.9]	4.6±1.9	4.5 [3.2–5.9]	0.23
PtGA (0–10), cm	5.3±2.8	5.0 [2.6–7.8]	4.9±2.6	4.7 [3.4–6.9]	0.51
ESR, mm/h	53.2±35.3	43 [24–78]	45.9±29.5	44 [19–59]	0.31
CRP, mg/dL	2.1±3.1	1.02 [0.22–2.88]	1.4±1.6	0.63 [0.18–2.16]	0.24
DAS28-ESR	5.3±1.5	5.1 [4.1–6.5]	5.1±1.1	5.1 [4.2–5.9]	0.53
SDAI	27.5±16.8	22.7 [14.8–39.8]	22.8±10.7	20.1 [15.2–28.0]	0.26
CDAI	25.4±15.1	20.1 [14.1–38.5]	21.4±10.3	19.0 [14.0–25.9]	0.26
HAQ-DI	1.1±0.8	1.0 [0.5–1.8]	1.0±0.6	1.0 [0.6–1.5]	0.74
RF positive, n(%)	45 (78.9) §		60 (85.7) §		0.35
ACPA positive, n(%)	47 (87.0) §		57 (83.8) §		0.80

Comparisons of baseline characteristics between the IFX and TCZ groups were performed by the Wilcoxon rank-sum test for continuous variables and Fisher’s exact test for comparisons between proportions. ACPA, anti-cyclic citrullinated protein/peptide antibody; CDAI, clinical disease activity index; CRP, C-reactive protein; DAS, disease activity score; DMARDs, disease-modifying antirheumatic drugs; ESR, erythrocyte sedimentation rate; HAQ-DI, health assessment questionnaire-disability index; IFX, infliximab; IQR, interquartile range; MTX, methotrexate; PhGA, physician/observer global assessment; PSL, prednisolone; PtGA, patient global assessment; RF, rheumatoid factor; SD, standard deviation; SDAI, simplified disease activity index; SJC, swollen joint count; TCZ, tocilizumab; TJC, tender joint count.

Asterisk (*) indicates P<0.05.

§Data are shown as number of patients (%).

### Clinical Effectiveness

**Fig 1:**
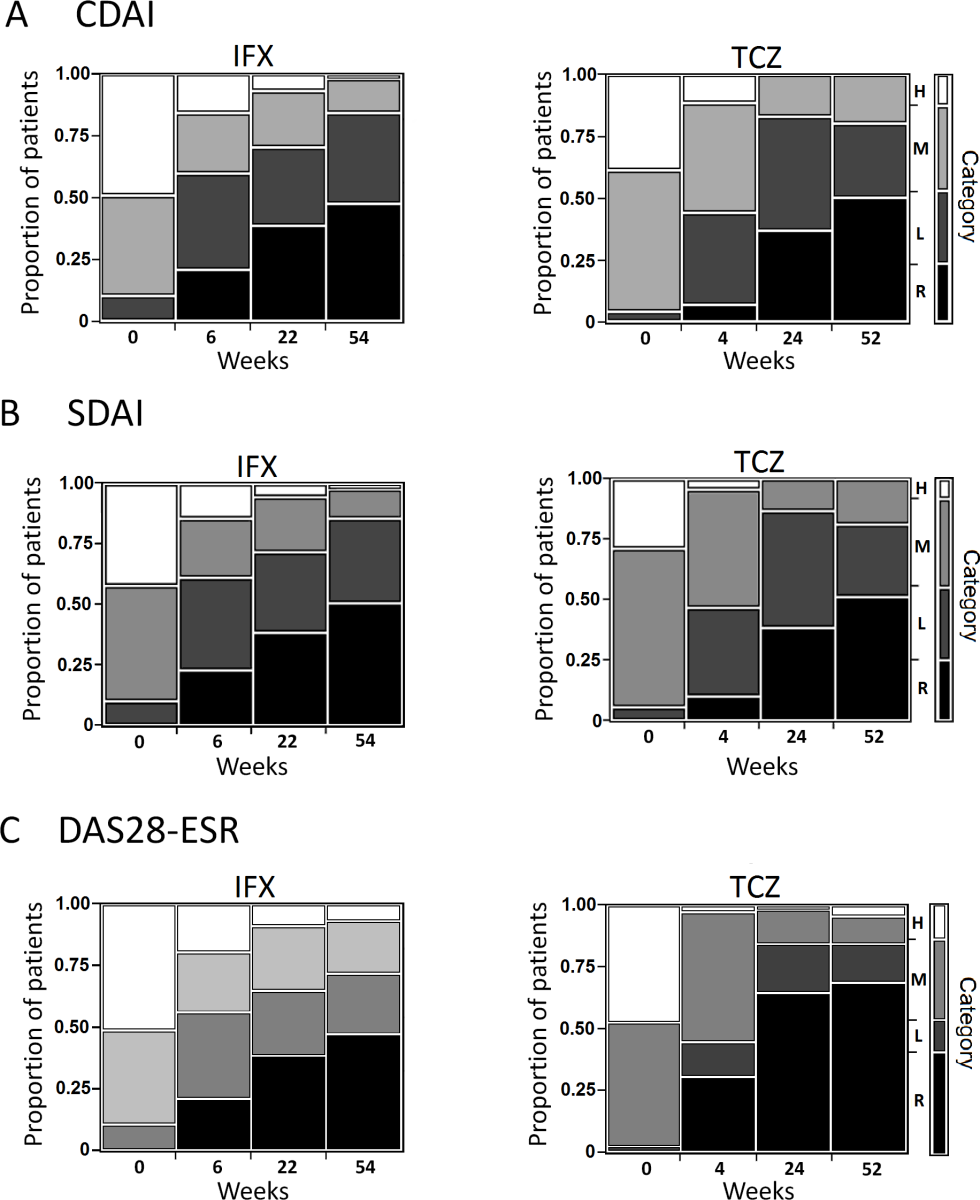
Categorical changes in clinical indices. (A) Clinical Disease Activity Index (CDAI), (B) Simplified Disease Activity Index (SDAI), and (C) disease activity score with 28 joint counts, erythrocyte sedimentation rate (DAS28-ESR), over 1-year treatment of infliximab (IFX) or tocilizumab (TCZ). H, high disease activity; M, moderate disease activity; L, low disease activity; R, remission.

As shown in Fig 1A–1C, the rates of CDAI and SDAI remissions at 1 year were comparable between the IFX and TCZ groups. The rate of DAS28-ESR remission in the TCZ group was significantly higher than that in the IFX group (68.6% vs 47.4%; P = 0.0189 by Fisher’s exact test). In the IFX group, we missed the disease activity data from 8 patients (14.0%) at week 54 because of the discontinuation of IFX (n = 7) and the lack of description in the medical records (n = 1), whereas in the TCZ group we missed the data from 4 patients (5.7%) at week 52 because of the discontinuation of TCZ (n = 3) and the lack of description in the medical records (n = 1). These missing data were replaced using the LOCF method.

### Safety and Drug Retention Rate

Within the first year, 7 patients discontinued IFX treatment because of Pneumocystis pneumonia (n = 1), infusion reaction (n = 1), moving (n = 1), inefficacy (n = 2), and improvement of symptoms (n = 2), while 3 discontinued TCZ treatment because of inefficacy (n = 1), and moving (n = 2). There was no significant difference in the retention rates at 1 year between the IFX group (91.0%) and the TCZ group (95.2%) (P = 0.0504).

### Baseline Predictors of Clinical Remission at 1 Year

**Fig 2:**
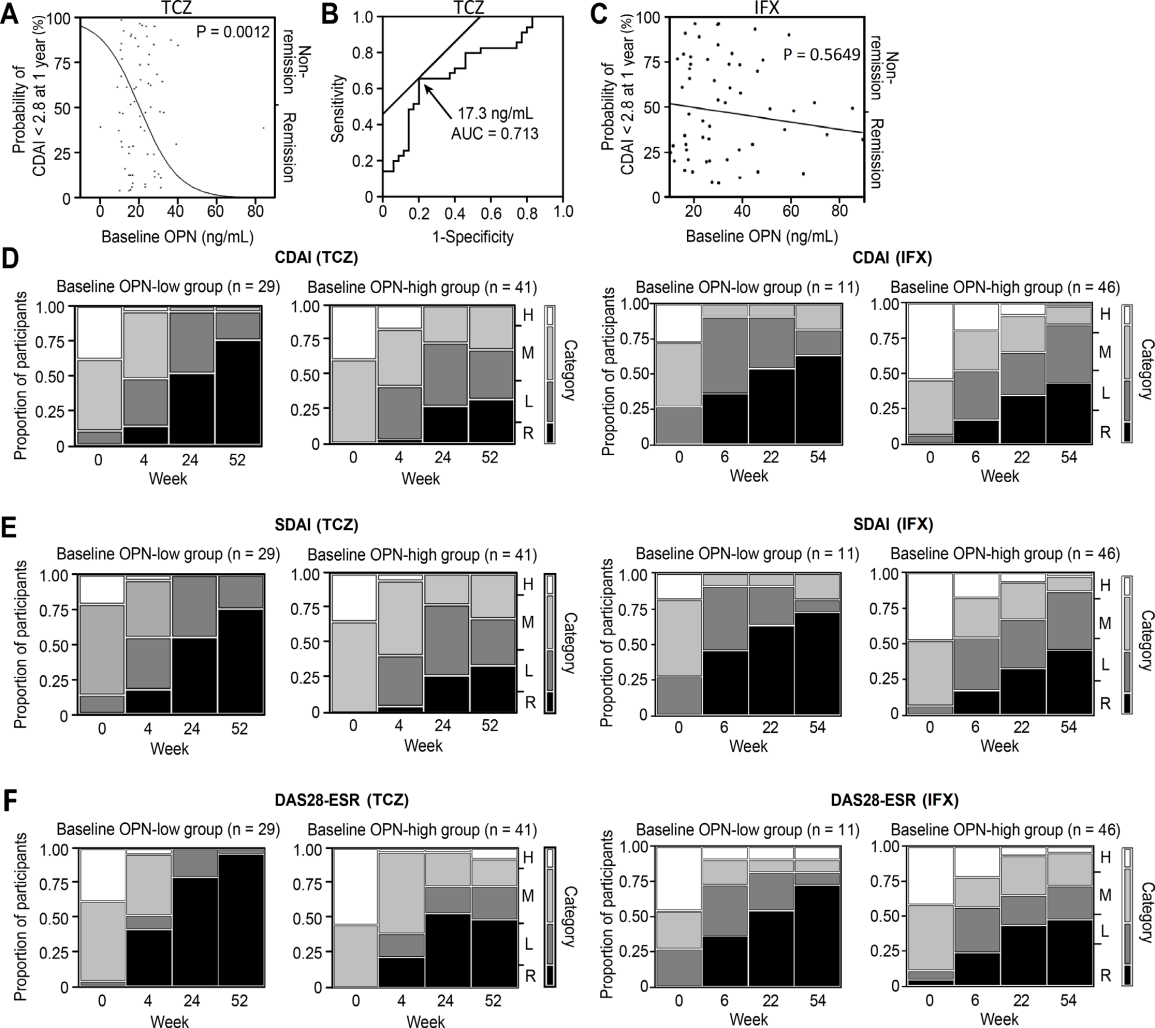
Predictive ability of osteopontin for clinical remission in patients with RA who received tocilizumab (TCZ) or infliximab (IFX). (A) Logistic regression analysis showing significant association of increasing baseline osteopontin (OPN) levels with decreasing predicted probability of achieving Clinical Disease Activity Index (CDAI) remission at 1 year in the TCZ (tocilizumab) group. (B) ROC curve showing a cut-off baseline OPN level of 17.3 ng/mL, discriminating between CDAI remission and non-remission at 1 year in the TCZ group, with a sensitivity of 66% and a specificity of 80%. (C) Logistic regression analysis showing no significant association of baseline OPN levels with predicted probability of achieving CDAI remission at 1 year in the IFX (infliximab) group. Categorical changes in clinical indices such as (D) CDAI, (E) Simplified Disease Activity Index (SDAI), and (F) disease activity score with 28 joint counts, erythrocyte sedimentation rate (DAS28-ESR) over 1-year treatment of TCZ or IFX, stratified by low or high baseline OPN levels (cut-off: 17.3 ng/mL). H, high disease activity; M, moderate disease activity; L, low disease activity; R, remission.

In the univariate analysis, no baseline variable was significantly associated with CDAI remission at 1 year in the IFX group (Table 2). HAQ-DI at baseline tended to be associated with CDAI remission at 1 year in the IFX group (P = 0.0706). Younger age (P = 0.0327) and lower OPN levels (P = 0.0012) at baseline (Fig 2A) were significantly associated with CDAI remission at 1 year in the TCZ group. Lower PSL dose (P = 0.0820) and lower CRP levels (P = 0.0603) at baseline tended to be associated with CDAI remission at 1 year in the TCZ group. In the multivariate analysis, lower OPN levels at baseline were significantly associated with CDAI remission at 1 year (P = 0.0178, OR 0.9145, 95%CI 0.8399–0.9857), whereas no variables in the IFX group were significantly associated. For SDAI remission at 1 year, the same predictive baseline factors as for CDAI were obtained in the multivariate analysis in the TCZ group, whereas no variables in the IFX group were significantly associated (S1 Table). Moreover, for DAS28-ESR remission at 1 year, lower serum levels of OPN and osteonectin at baseline were obtained as significant predictive factors in the multivariate analysis in the TCZ group, whereas men were obtained as a significant predictive factor in the IFX group (S2 Table). Only OPN in the TCZ group was common among the multivariate analyses of baseline factors for CDAI, SDAI and DAS28-ESR remission at 1 year. Baseline serum OPN levels between CDAI remission and non-remission patients at 1 year were shown in S1 Fig. Baseline levels of OPN was significantly higher in the patients with CDAI remission (n = 35) than in those with CDAI non-remission (n = 35) (P = 0.0022). Furthermore, when we excluded the patients without concomitant MTX, baseline levels of OPN was also significantly higher in the patients with CDAI remission (n = 26) than in those with CDAI non-remission (n = 27) (P = 0.0198) (S2 Fig). Correlations between serum OPN levels and other variables at baseline are in S3 Table.

**Table 2:** Univariate and multivariate logistic regression analyses of baseline factors for CDAI remission at 1 year.

	Univariate analysis				Multivariate analysis			
	IFX		TCZ		IFX		TCZ	
	OR (95% CI)	P	OR (95% CI)	P	OR (95% CI)	P	OR (95% CI)	P
Age, years	0.9715 (0.9306–1.0107)	0.1543	0.9582 (0.9175–0.9966)	0.0327*			0.9873 (0.9403–1.0352)	0.5953
Sex (Men/Women)	1.8571 (0.4692–8.0879)	0.3779	0.7742 (0.1768–3.1955)	0.7208				
Disease duration, years	0.9668 (0.9046–1.0273)	0.2797	0.9802 (0.9077–1.0528)	0.5820				
PSL dose, mg/day	0.9142 (0.7435–1.0430)	0.2068	0.8699 (0.7117–1.0162)	0.0820			0.8956 (0.7130–1.1059)	0.3083
MTX dose, mg/week	1.0786 (0.8823–1.3456)	0.4617	1.0309 (0.9181–1.1603)	0.6058				
Other DMARDs use	1.1250 (0.1923–6.5829)	0.8915	0.4697 (0.0619–2.5833)	0.3890				
CDAI	0.9746 (0.9369–1.0100)	0.1604	0.9693 (0.9192–1.0162)	0.1998				
CRP, mg/dL	0.9146 (0.7281–1.0929)	0.3388	0.7420 (0.5210–1.0125)	0.0603			0.9070 (0.6202–1.3117)	0.6012
RF (positive/negative)	0.5714 (0.1489–2.0581)	0.3917	1.0000 (0.2538–3.9405)	1.0000				
ACPA (positive/negative)	0.7159 (0.1281–3.6213)	0.6831	1.3846 (0.3750–5.3127)	0.6223				
HAQ-DI	0.5419 (0.2609–1.0514)	0.0706	0.5670 (0.2551–1.1947)	0.1371				
IFN-γ, pg/mL	1.0340 (0.8011–1.3831)	0.7782	0.9272 (0.7210–1.1194)	0.4391				
IL-1β, pg/mL	1.1177 (0.7548–1.8222)	0.5702	0.4662 (0.1485–1.2493)	0.1329				
IL-2, pg/mL	1.2195 (0.7013–2.5555)	0.4812	0.7590 (0.2690–1.7876)	0.5281				
IL-6, pg/mL	0.9900 (0.9485–1.0298)	0.6174	0.9841 (0.9399–1.0080)	0.2286				
IL-8, pg/mL	1.0006 (0.9988–1.0040)	0.4980	0.9971 (0.9831–1.0095)	0.6428				
IL-10, pg/mL	1.0044 (0.9724–1.0427)	0.7717	1.0440 (0.9430–1.1952)	0.4147				
IL-17, pg/mL	1.0396 (0.2586–4.1254)	0.9545	0.3986 (0.0396–1.9271)	0.2724				
TNF-α, pg/mL	0.9739 (0.8397–1.0321)	0.4107	0.9368 (0.8003–1.0425)	0.2540				
sICAM-1, ng/mL	0.9945 (0.9747–1.0133)	0.5627	0.9790 (0.9482–1.0040)	0.1033				
BAP, ng/mL	0.9911 (0.9620–1.0167)	0.4958	0.9954 (0.9659–1.0242)	0.7460				
Osteonectin, ng/mL	1.0134 (0.9337–1.1010) (per 100 units)	0.7484	0.9351 (0.8393–1.0348) (per 100 units)	0.1958				
OPN, ng/mL	0.9917 (0.9623–1.0202)	0.5649	0.9044 (0.8377–0.9654)	0.0012*			0.9145 (0.8399–0.9857)	0.0178*

Baseline factors with P values less than 0.1 in univariate analysis were entered into multivariate analysis.

Asterisks (*) indicate P<0.05 by the likelihood ratio test. ACPA, anti-cyclic citrullinated protein/peptide antibody; BAP, bone alkaline phosphatase; CDAI, Clinical Disease Activity Index; CI, confidence intervals; CRP, C-reactive protein; ESR, erythrocyte sedimentation rate; HAQ-DI, health assessment questionnaire disability index; IFN, interferon; IFX, infliximab; IL, interleukin; MTX, methotrexate; OR, odds ratio; OPN, osteopontin; PSL, prednisolone; RF, rheumatoid factor; sICAM-1, soluble intercellular adhesion molecule-1; TCZ, tocilizumab; TNF, tumor necrosis factor.

In the TCZ group, the ROC curve showed a cut-off OPN level of 17.3 ng/mL with the area under the curve of 0.713, which discriminated patients with CDAI remission and non-remission with a sensitivity of 66% and a specificity of 80% (Fig 2B), while in the IFX group. As for SDAI and DAS28-ESR remission, in a similar fashion, the areas under the curve were 0.701 and 0.799, respectively, with a sensitivity of 64% and 60%, respectively, and with a specificity of 80% and 95%, respectively (S3 and S4 Figs). When we excluded the patients in the TCZ group who did not receive concomitant MTX and analyzed the patients in the TCZ group who received concomitant MTX (n = 53), we obtained the similar results that baseline levels of OPN still had a significant predictive value for clinical remission at 1 year (analysis of CDAI remission shown in S5 Fig; analysis of SDAI and DAS28-ESR remission also had significant P values [data not shown]).

The numbers of patients with baseline OPN levels of ≤17.3 ng/mL (OPN-low) and >17.3 ng/mL (OPN-high) were 29 and 41, respectively. The number needed to treat (test) (NNT) of the cut-off OPN level to predict CDAI remission is 2.26.

The CDAI, SDAI and DAS28-ESR category changes in the OPN-low and OPN-high patients in the TCZ group or the IFX group are presented in Fig 2D–2F. A significantly higher proportion of patients in the OPN-low group achieved CDAI, SDAI, and DAS28-ESR remission compared with the patients in the OPN-high group (76% vs 32%, P = 0.0006; 76% vs 34%, P = 0.0007; 97% vs 49%, P<0.0001, respectively by Fisher’s exact test). The baseline characteristics of the patients were not significantly different between the OPN-low and OPN-high groups (data not shown).

## Discussion

The main result of our single-center prospective study is the identification of predicting baseline factors of response to IFX or TCZ for biologic-naïve patients with RA. In the multivariate analysis, lower serum OPN levels at baseline were significantly associated with achieving clinical remissions 1 year after initiating TCZ treatment, whereas none of the baseline factors were significantly associated with achieving clinical remissions after IFX treatment.

Between the IFX and TCZ groups, there were no significant differences in the clinical effectiveness or in the baseline characteristics other than MTX use. For the patients who are intolerant of MTX, TCZ is preferably selected[6]. In our study, all of the patients used MTX in the IFX group, whereas three fourths of the patients used MTX in the TCZ group. Therefore, the difference in the baseline MTX use might cause differences in the predictive factors of clinical remission between the two groups. However, in the TCZ group there were no significant differences in the baseline characteristics of the patients with or without MTX. Moreover, when we compared the IFX group and the MTX-using patients in the TCZ group, there were no significant differences in the baseline characteristics between the groups. Regardless of the MTX use at baseline, the patients in the TCZ group showed the similar significant associations of baseline OPN levels with achieving clinical remission at 1 year.

The association of baseline biomarkers with the response to TCZ has not been well studied. Although some previous studies reported that baseline RF positivity predicts better response to TCZ[7, 8], our study did not demonstrate the significant association between RF and response to TCZ. While we have previously reported that soluble IL-6 receptors (sIL-6R) predict clinical remission (defined by the criteria of CDAI, SDAI, and DAS28-ESR) at week 24 in biologic-naïve patients with RA[9], OPN that we report this time has a higher predictive value than sIL-6R (AUC for predicting SDAI remission by sIL-6R and OPN, 0.63 and 0.70, respectively). We determined that OPN is a predictor of clinical remission with TCZ treatment among many clinical and biological factors including 12 proteins measured by an electrochemiluminescence assay that has lower limits of detection sensitivity than conventional ELISA. Moreover, our study required evaluation only at baseline to predict clinical remission, although most of the previous studies required changes from baseline levels to subsequent visits[10, 11].

OPN is an extracellular matrix protein that is overexpressed in the synovial membrane, fibroblast-like synoviocytes and synovial CD4+T cells of RA patients; this protein has proinflammatory cytokine-like properties by binding to several integrins and CD44[12]. Elevated blood levels of secreted OPN were found in patients with RA[13] and the *secreted phosphoprotein 1 (SPP1)* gene, which encodes OPN, has been identified as a new RA susceptibility gene[14]. OPN appears to act as a paracrine and autocrine amplification factor for cytokine release in the joints, inducing the production of IL-6[15]. OPN-deficient mice exhibit reduced disease in arthritis models not by altering TNF-α levels but by suppressing angiogenesis in the joints[16], and angiogenesis is enhanced by IL-6 signaling instead of by TNF-α[17]. Actually, OPN was significantly correlated with CRP and IL-6, but not with TNF-α in our study. In addition, average levels of inflammation response markers (CRP and ESR) and average scores of clinical activity indices were lower in the TCZ group than in the IFX group, although there were not significant differences between the groups. Along with these facts, we believe that a better response to TCZ is seen in the patients with less marked inflammation because less inflammation is easier to treat. This might facilitate understanding our findings that OPN is related to the response to TCZ treatment but not to IFX.

As for measurements of OPN levels in the blood, although some previous studies, along with our study, adopted measurements of serum OPN[8, 11], it has been reported that plasma OPN is more stable than serum OPN and that measurements of plasma OPN is more reliable[15]. In the case of a replication study to confirm our results, plasma OPN may have to be measured.

Our study demonstrated that low serum OPN levels predict clinical remission after 1-year TCZ treatment but not IFX treatment. Our prediction model provided insights into how to optimize the choice of biologics and warrants external validation in other cohorts.

## Supporting Information

S1 Table Univariate and multivariate logistic regression analyses of baseline factors for SDAI remission at 1 year. (DOCX)

S2 Table Univariate and multivariate logistic regression analyses of baseline factors for DAS28-ESR remission at 1 year. (DOCX)

S3 Table Correlations between serum OPN levels (ng/mL) and other variables at baseline in all of the patients (n = 127). (DOCX)

S4 Table Baseline characteristics of the patients who received IFX and those who received TCZ with concomitant MTX. (DOCX)

S1 Fig Comparison of baseline serum levels of osteopontin (OPN) between patients with CDAI remission (n = 35) and with non-remission (n = 35) who received TCZ. (DOCX)

S2 Fig Comparison of baseline serum levels of osteopontin (OPN) between patients with CDAI remission (n = 26) and with non-remission (n = 27) who received TCZ with concomitant MTX. (DOCX)

S3 Fig Predictive ability of OPN for SDAI remission in patients with RA who received TCZ. (DOCX)

S4 Fig Predictive ability of OPN for DAS28-ESR remission in patients with RA who received TCZ. (DOCX)

S5 Fig Predictive ability of OPN for CDAI remission in patients with RA who received TCZ with concomitant MTX. (DOCX)
