# Correction: Baseline Serum Osteopontin Levels Predict the Clinical Effectiveness of Tocilizumab but Not Infliximab in Biologic-Naïve Patients with Rheumatoid Arthritis: A Single-Center Prospective Study at 1 Year (the Keio First-Bio Cohort Study)

**DOI:** 10.1371/journal.pone.0152341

**Published:** 2016-03-21

**Authors:** Keisuke Izumi, Yuko Kaneko, Misato Hashizume, Keiko Yoshimoto, Tsutomu Takeuchi

The x-axis in Panel A within S5 Fig is labeled incorrectly. It should read: 10, 20, 30, 40. Please view the correct S5 Fig below.

## Supporting Information

S5 Fig Predictive ability of OPN for CDAI remission in patients with RA who received TCZ with concomitant MTX. (TIF)
