# Supplementary material for: Baseline Serum Osteopontin Levels Predict the Clinical Effectiveness of Tocilizumab but Not Infliximab in Biologic-Naïve Patients with Rheumatoid Arthritis: A Single-Center Prospective Study at 1 Year (the Keio First-Bio Cohort Study)
Source: PLoS One. 2015 Dec 23;10(12):e0145468. doi: 10.1371/journal.pone.0145468 (PMC4689361; doi:10.1371/journal.pone.0145468)
Supplement: S1 Table — (DOCX) [file pone.0145468.s001.docx]

**S1 Table.** **Univariate and multivariate logistic regression analyses of baseline factors for SDAI remission at 1 year**

|  | **Univariate analysis** | | | | **Multivariate analysis** | | | |
| --- | --- | --- | --- | --- | --- | --- | --- | --- |
|  | **IFX** | | **TCZ** | | **IFX** | | **TCZ** | |
|  | OR (95% CI) | P | OR (95% CI) | P | OR (95% CI) | P | OR (95% CI) | P |
| Age, years | 0.9637 (0.9306-1.0107) | 0.0740 | 0.9565 (0.9153-0.9951) | 0.0270* | 0.9759 (0.9305-1.0202) | 0.2834 | 0.9841 (0.9369-1.0320) | 0.5086 |
| Sex (Men/Women) | 1.5652 (0.3956-6.8045) | 0.5238 | 0.7250 (0.1655-2.9925) | 0.6533 |  |  |  |  |
| Disease duration, years | 0.9616 (0.8999-1.0217) | 0.2082 | 0.9786 (0.9064-1.0511) | 0.5511 |  |  |  |  |
| PSL dose, mg/day | 0.8981 (0.7263-1.0300) | 0.1408 | 0.8597 (0.7014-1.0065) | 0.0061 |  |  | 0.8833 (0.7030-1.0894) | 0.2494 |
| MTX dose, mg/week | 0.9657 (0.7474-1.2387) | 0.7800 | 1.0295 (0.9167-1.1583) | 0.6227 |  |  |  |  |
| Other DMARDs use | 0.9615 (0.1643-5.6258) | 0.9638 | 0.4412 (0.0582-2.4267) | 0.3502 |  |  |  |  |
| SDAI | 0.9675 (0.9324-0.9997) | 0.0478* | 0.9799 (0.9342-1.0243) | 0.3706 | 0.9811 (0.9398-1.0225) | 0.3635 |  |  |
| CRP, mg/dL | 0.8800 (0.6907-1.0581) | 0.1851 | 0.7497 (0.5298-1.0203) | 0.0674 |  |  | 0.9197 (0.6317-1.3293) | 0.6524 |
| RF (positive/negative) | 0.6832 (0.1785-2.4595) | 0.5601 | 1.0690 (0.2713-4.2134) | 0.9222 |  |  |  |  |
| ACPA (positive/negative) | 1.5152 (0.3022-8.4029) | 0.6095 | 1.4308 (0.3882-5.4823) | 0.5872 |  |  |  |  |
| HAQ-DI | 0.4713 (0.2220-0.9236) | 0.0278* | 0.5758 (0.2560-1.2121) | 0.1475 | 0.6652 (0.2706-1.5580) | 0.3489 |  |  |
| IFN-γ, pg/mL | 1.0055 (0.7719-1.3242) | 0.9633 | 0.9169 (0.7090-1.1081) | 0.3793 |  |  |  |  |
| IL-1β, pg/mL | 1.0687 (0.7193-1.7057) | 0.7323 | 0.4602 (0.1475-1.2303) | 0.1251 |  |  |  |  |
| IL-2, pg/mL | 1.1477 (0.6595-2.3279) | 0.6208 | 0.7019 (0.2399-1.6573) | 0.4239 |  |  |  |  |
| IL-6, pg/mL | 0.9806 (0.9377-1.0202) | 0.3348 | 0.9843 (0.9409-1.0079) | 0.2261 |  |  |  |  |
| IL-8, pg/mL | 1.0005 (0.9987-1.0038) | 0.5726 | 0.9967 (0.9825-1.0090) | 0.5892 |  |  |  |  |
| IL-10, pg/mL | 1.0022 (0.9699-1.0396) | 0.8846 | 1.0405 (0.9400-1.1905) | 0.4514 |  |  |  |  |
| IL-17, pg/mL | 0.8080 (0.1954-3.1501) | 0.7542 | 0.4324 (0.0474-2.0291) | 0.3040 |  |  |  |  |
| TNF-α, pg/mL | 0.9696 (0.8282-1.0287) | 0.3472 | 0.9333 (0.7963-1.0394) | 0.2299 |  |  |  |  |
| sICAM-1, ng/mL | 0.9913 (0.9713-1.0101) | 0.3662 | 0.9799 (0.9499-1.0046) | 0.1138 |  |  |  |  |
| BAP, ng/mL | 0.9874 (0.9576-1.0130) | 0.3382 | 0.9930 (0.9633-1.0217) | 0.6258 |  |  |  |  |
| Osteonectin, ng/mL | 1.0030 (0.9240-1.0892) (per 100 units) | 0.9419 | 0.9264 (0.8307-1.0256) (per 100 units) | 0.1423 |  |  |  |  |
| OPN, ng/mL | 0.9851 (0.9550-1.0134) | 0.3010 | 0.9099 (0.8443-0.9697) | 0.0019* |  |  | 0.9209 (0.8470-0.9917) | 0.0280* |

Baseline factors with P values less than 0.1 in univariate analysis were entered into multivariate analysis. Asterisks (*) indicate P<0.05 by the likelihood ratio test.

ACPA, anti-cyclic citrullinated protein/peptide antibody; BAP, bone alkaline phosphatase; CI, confidence intervals; CRP, C-reactive protein; HAQ-DI, health assessment questionnaire disability index; IFN, interferon; IFX, infliximab; IL, interleukin; MTX, methotrexate; OR, odds ratio; OPN, osteopontin; PSL, prednisolone; RF, rheumatoid factor; SDAI, Simplified Disease Activity Index; sICAM-1, soluble intercellular adhesion molecule-1; TCZ, tocilizumab; TNF, tumor necrosis factor.
