# Supplementary material for: Baseline Serum Osteopontin Levels Predict the Clinical Effectiveness of Tocilizumab but Not Infliximab in Biologic-Naïve Patients with Rheumatoid Arthritis: A Single-Center Prospective Study at 1 Year (the Keio First-Bio Cohort Study)
Source: PLoS One. 2015 Dec 23;10(12):e0145468. doi: 10.1371/journal.pone.0145468 (PMC4689361; doi:10.1371/journal.pone.0145468)
Supplement: S2 Table — (DOCX) [file pone.0145468.s002.docx]

**S2 Table.** **Univariate and multivariate logistic regression analyses of baseline factors for DAS28-ESR remission at 1 year**

|  | **Univariate analysis** | | | | **Multivariate analysis** | | | |
| --- | --- | --- | --- | --- | --- | --- | --- | --- |
|  | **IFX** | | **TCZ** | | **IFX** | | **TCZ** | |
|  | OR (95% CI) | P | OR (95% CI) | P | OR (95% CI) | P | OR (95% CI) | P |
| Age, years | 0.9694 (0.9282-1.0087) | 0.1270 | 0.9674 (0.9230-1.0090) | 0.1257 |  |  |  |  |
| Sex (Men/Women) | 5.8947 (1.3045-41.988) | 0.0198* | 0.9048 (0.2140-4.6402) | 0.8956 | 6.6910 (1.2270-59.185) | 0.0267* |  |  |
| Disease duration, years | 0.9523 (0.8877-1.0132) | 0.1248 | 0.9564 (0.8857-1.0298) | 0.2312 |  |  |  |  |
| PSL dose, mg/day | 0.9142 (0.7435-1.0430) | 0.2068 | 0.9747 (0.8432-1.1406) | 0.7277 |  |  |  |  |
| MTX dose, mg/week | 1.0433 (0.8131-1.3479) | 0.7348 | 1.0466 (0.9240-1.1851) | 0.4692 |  |  |  |  |
| Other DMARDs use | 1.1250 (0.1923-6.5859) | 0.8915 | 0.9091 (0.1631-6.9430) | 0.9167 |  |  |  |  |
| DAS28-ESR | 0.7259 (0.4947-1.0314) | 0.0744 | 0.4911 (0.2771-0.8102) | 0.0046* | 0.8050 (0.4782-1.3196) | 0.3893 | 1.0022 (0.4133-2.6284) | 0.9962 |
| CRP, mg/dL | 0.8811 (0.6866-1.0620) | 0.1978 | 0.7746 (0.5610-1.0570) | 0.1065 |  |  |  |  |
| RF (positive/negative) | 0.3654 (0.0868-1.3378) | 0.1298 | 0.5857 (0.0822-2.6925) | 0.5125 |  |  |  |  |
| ACPA (positive/negative) | 0.9200 (0.1967-4.3016) | 0.9131 | 0.8125 (0.1635-3.1915) | 0.7751 |  |  |  |  |
| HAQ-DI | 0.4452 (0.2047-0.8833) | 0.0198* | 0.2533 (0.0918-0.6083) | 0.0016* | 0.2053 (0.0098-3.7369) | 0.2847 | 0.3205 (0.0621-1.3589) | 0.1247 |
| IFN-γ, pg/mL | 1.0371 (0.8046-1.3913) | 0.7592 | 1.1245 (0.9011-1.6202) | 0.3500 |  |  |  |  |
| IL-1β, pg/mL | 1.0509 (0.7005-1.6322) | 0.7959 | 0.2508 (0.0726-0.7251) | 0.0096* |  |  | 0.2268 (0.0436-1.0112) | 0.0517 |
| IL-2, pg/mL | 1.1722 (0.6731-2.3604) | 0.5663 | 0.6949 (0.2611-1.7039) | 0.4052 |  |  |  |  |
| IL-6, pg/mL | 0.9859 (0.9434-1.0256) | 0.4835 | 0.9454 (0.8902-0.9901) | 0.0074* |  |  | 0.9917 (0.9263-1.0452) | 0.7704 |
| IL-8, pg/mL | 1.0006 (0.9988-1.0041) | 0.4962 | 1.0035 (0.9906-1.0225) | 0.6261 |  |  |  |  |
| IL-10, pg/mL | 0.7681 (0.4864-0.9888) | 0.0177* | 1.0039 (0.9058-1.1478) | 0.9422 | 0.8775 (0.5577-1.0058) | 0.0972 |  |  |
| IL-17, pg/mL | 0.9534 (0.2333-3.7422) | 0.9441 | 0.1319 (0.0087-0.8819) | 0.0343* |  |  | 0.2996 (0.0040-12.470) | 0.5375 |
| TNF-α, pg/mL | 0.9352 (0.7381-1.0208) | 0.2106 | 0.9712 (0.8659-1.0823) | 0.5605 |  |  |  |  |
| sICAM-1, ng/mL | 0.9901 (0.9695-1.0091) | 0.3091 | 0.9881 (0.9634-1.0126) | 0.3301 |  |  |  |  |
| BAP, ng/mL | 0.9988 (0.9722-1.0249) | 0.9226 | 1.0023 (0.9732-1.0376) | 0.8807 |  |  |  |  |
| Osteonectin, ng/mL | 0.9815 (0.9027-1.0651) (per 100 units) | 0.6530 | 0.8966 (0.7981-0.9995) (per 100 units) | 0.0490* |  |  | 0.8410 (0.7009-0.9871) (per 100 units) | 0.0337* |
| OPN, ng/mL | 0.9866 (0.9563-1.0151) | 0.3570 | 0.8702 (0.7939-0.9390) | <0.0001* |  |  | 0.8710 (0.7771-0.9588) | 0.0037* |

Baseline factors with P values less than 0.1 in univariate analysis were entered into multivariate analysis. Asterisks (*) indicate P<0.05 by the likelihood ratio test.

ACPA, anti-cyclic citrullinated protein/peptide antibody; BAP, bone alkaline phosphatase; CI, confidence intervals; CRP, C-reactive protein; DAS28-ESR, disease activity score 28-erythrocyte sedimentation rate; HAQ-DI, health assessment questionnaire disability index; IFN, interferon; IFX, infliximab; IL, interleukin; MTX, methotrexate; OR, odds ratio; OPN, osteopontin; PSL, prednisolone; RF, rheumatoid factor; sICAM-1, soluble intercellular adhesion molecule-1; TCZ, tocilizumab; TNF, tumor necrosis factor.
