# Supplementary material for: Baseline Serum Osteopontin Levels Predict the Clinical Effectiveness of Tocilizumab but Not Infliximab in Biologic-Naïve Patients with Rheumatoid Arthritis: A Single-Center Prospective Study at 1 Year (the Keio First-Bio Cohort Study)
Source: PLoS One. 2015 Dec 23;10(12):e0145468. doi: 10.1371/journal.pone.0145468 (PMC4689361; doi:10.1371/journal.pone.0145468)
Supplement: S3 Table — (DOCX) [file pone.0145468.s003.docx]

**S3 Table. Correlations between serum OPN levels (ng/mL) and other variables at baseline in all of the patients (n=127)**

| **Baseline variables** | **Spearman's rank correlation coefficient (ρ)** | **P value** |
| --- | --- | --- |
| Age, years | 0.3213 | 0.0002* |
| Disease duration, years | 0.1837 | 0.0387* |
| PSL dose, mg/day | 0.0814 | 0.3632 |
| MTX dose, mg/week | 0.1523 | 0.0874 |
| CDAI | 0.3549 | <0.0001* |
| CRP, mg/dL | 0.4260 | <0.0001* |
| HAQ-DI | 0.2539 | 0.0041* |
| IFN-γ, pg/mL | 0.2059 | 0.0202* |
| IL-1β, pg/mL | 0.3351 | 0.0001* |
| IL-2, pg/mL | 0.1246 | 0.1628 |
| IL-6, pg/mL | 0.4090 | <0.0001* |
| IL-8, pg/mL | 0.3552 | <0.0001* |
| IL-10, pg/mL | 0.2236 | 0.0115* |
| IL-17, pg/mL | 0.2278 | 0.0100* |
| TNF-α, pg/mL | 0.0111 | 0.9013 |
| sICAM-1, ng/mL | 0.3066 | 0.0006* |
| BAP, ng/mL | -0.0338 | 0.7058 |
| Osteonectin, ng/mL | 0.3439 | <0.0001* |

Asterisks (*) indicate P<0.05. ACPA, anti-cyclic citrullinated protein/peptide antibody; BAP, bone alkaline phosphatase; CDAI, Clinical Disease Activity Index; CRP, C-reactive protein; HAQ-DI, health assessment questionnaire disability index; IFN, interferon; IL, interleukin; MTX, methotrexate; PSL, prednisolone; RF, rheumatoid factor; sICAM-1, soluble intercellular adhesion molecule-1; TNF, tumor necrosis factor.
