# Supplementary material for: Baseline Serum Osteopontin Levels Predict the Clinical Effectiveness of Tocilizumab but Not Infliximab in Biologic-Naïve Patients with Rheumatoid Arthritis: A Single-Center Prospective Study at 1 Year (the Keio First-Bio Cohort Study)
Source: PLoS One. 2015 Dec 23;10(12):e0145468. doi: 10.1371/journal.pone.0145468 (PMC4689361; doi:10.1371/journal.pone.0145468)
Supplement: S4 Table — (DOCX) [file pone.0145468.s004.docx]

**S4 Table. Baseline characteristics of the patients who received IFX and those who received TCZ with concomitant MTX**

|  | **IFX (n=57)** | | **TCZ + MTX (n=53)** | | **P value** |
| --- | --- | --- | --- | --- | --- |
|  | **Mean±SD** | **Median [IQR]** | **Mean±SD** | **Median [IQR]** |  |
| Age, years | 55.8±13.5 | 56.0 [47.0-67.0] | 56.1±12.4 | 59.0 [48.5-64.0] | 0.98 |
| Women, n (%) | 47 (82.5) § |  | 46 (86.8) § |  | 0.60 |
| Duration, years | 7.7±8.8 | 3.1 [0.5-13.6] | 4.7±3.9 | 3.9 [1.5-7.5] | 0.68 |
| PSL use, n (%) | 11 (19.3) § |  | 17 (32.1) § |  | 0.13 |
| PSL dose in PSL-use patients, mg/day | 1.7±4.7 | 0 [0-0] | 1.5±2.5 | 0 [0-3] | 0.29 |
| MTX dose, mg/week | 8.5±2.1 | 8 [8-9] | 8.5±2.1 | 8 [8-10] | 0.71 |
| Other DMARDs use, n (%) | 6 (10.5) § |  | 2 (3.8) § |  | 0.27 |
| SJC (28 joints) | 8.3±6.2 | 7 [4-13] | 6.2±4.5 | 5 [3-8] | 0.11 |
| TJC (28 joints) | 6.8±6.9 | 5 [1-11] | 5.5±4.3 | 5 [3-7] | 0.87 |
| PhGA (0-10), cm | 5.0±2.3 | 5.0 [3.0-6.9] | 4.4±1.8 | 4.5 [3.1-5.6] | 0.11 |
| PtGA (0-10), cm | 5.3±2.8 | 5.0 [2.6-7.8] | 4.7±2.6 | 4.5 [3.0-6.5] | 0.30 |
| ESR, mm/h | 53.2±35.3 | 43 [24-78] | 43.4±30.5 | 41 [18-56] | 0.15 |
| CRP, mg/dL | 2.1±3.1 | 1.02 [0.22-2.88] | 1.3±1.5 | 0.47 [0.12-2.08] | 0.10 |
| DAS28-ESR | 5.3±1.5 | 5.1 [4.1-6.5] | 5.0±1.2 | 5.0 [4.0-5.9] | 0.38 |
| SDAI | 27.5±16.8 | 22.7 [14.8-39.8] | 22.1±11.4 | 19.8 [13.7-26.4] | 0.13 |
| CDAI | 25.4±15.1 | 20.1 [14.1-38.5] | 20.8±10.9 | 18.5 [13.3-24.8] | 0.16 |
| HAQ-DI | 1.1±0.8 | 1.0 [0.5-1.8] | 1.0±0.6 | 1.0 [0.5-1.3] | 0.49 |
| RF positive, n(%) | 45 (78.9) § |  | 47 (88.7) § |  | 0.20 |
| ACPA positive, n(%) | 47 (87.0) § |  | 43 (84.3) § |  | 1.00 |

Comparisons of baseline characteristics between infliximab and tocilizumab groups were performed by the Wilcoxon rank-sum test for continuous variables and Fisher’s exact test for comparisons between proportions.

§Data are shown as number of patients (%).

ACPA, anti-cyclic citrullinated protein/peptide antibody; CDAI, clinical disease activity index; CRP, C-reactive protein; DAS, disease activity score; DMARDs, disease-modifying antirheumatic drugs; ESR, erythrocyte sedimentation rate; HAQ-DI, health assessment questionnaire-disability index; IFX, infliximab; IQR, interquartile range; MTX, methotrexate; PhGA, physician/observer global assessment; PSL, prednisolone; PtGA, patient global assessment; RF, rheumatoid factor; SD, standard deviation; SDAI, simplified disease activity index; SJC, swollen joint count; TCZ, tocilizumab; TJC, tender joint count.
