# Supplementary material for: Baseline Serum Osteopontin Levels Predict the Clinical Effectiveness of Tocilizumab but Not Infliximab in Biologic-Naïve Patients with Rheumatoid Arthritis: A Single-Center Prospective Study at 1 Year (the Keio First-Bio Cohort Study)
Source: PLoS One. 2015 Dec 23;10(12):e0145468. doi: 10.1371/journal.pone.0145468 (PMC4689361; doi:10.1371/journal.pone.0145468)

**S2 Fig. Comparison of baseline serum levels of osteopontin (OPN) between patients with CDAI remission (n=26) and with non-remission (n=27) who received TCZ with concomitant MTX**


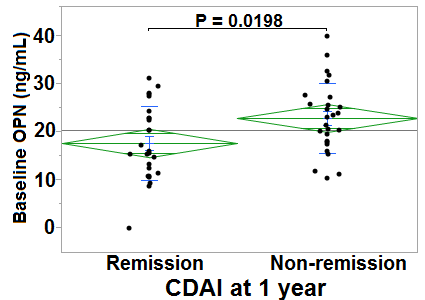

Supplement: S2 Fig — (DOCX) [file pone.0145468.s006.docx]
