# Supplementary material for: Baseline Serum Osteopontin Levels Predict the Clinical Effectiveness of Tocilizumab but Not Infliximab in Biologic-Naïve Patients with Rheumatoid Arthritis: A Single-Center Prospective Study at 1 Year (the Keio First-Bio Cohort Study)
Source: PLoS One. 2015 Dec 23;10(12):e0145468. doi: 10.1371/journal.pone.0145468 (PMC4689361; doi:10.1371/journal.pone.0145468)
Supplement: S5 Fig — (DOCX) [file pone.0145468.s009.docx]

**S5 Fig. Predictive ability of OPN for CDAI remission in patients with RA who received TCZ with concomitant MTX**

(A) Logistic regression analysis showing association of increasing baseline osteopontin (OPN) levels with decreasing predicted probability of achieving Clinical Disease Activity Index (CDAI) remission at 1 year. (B) ROC curve showing a cut-off baseline OPN level of 17.3 ng/mL, discriminating between CDAI remission and non-remission at 1 year, with a sensitivity of 58% and a specificity of 81%.

**
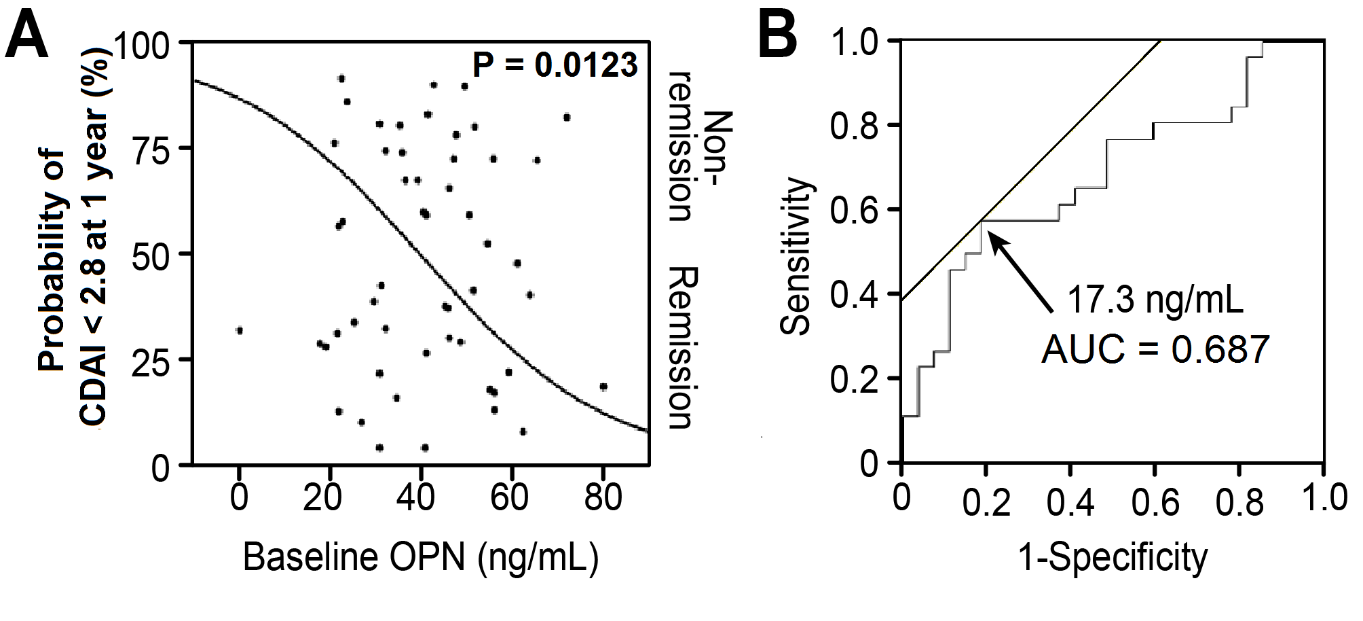
**
