# Supplementary figures and images for: Correction: Baseline Serum Osteopontin Levels Predict the Clinical Effectiveness of Tocilizumab but Not Infliximab in Biologic-Naïve Patients with Rheumatoid Arthritis: A Single-Center Prospective Study at 1 Year (the Keio First-Bio Cohort Study)
Source: PLoS One. 2016 Mar 21;11(3):e0152341. doi: 10.1371/journal.pone.0152341 (PMC4801414; doi:10.1371/journal.pone.0152341)

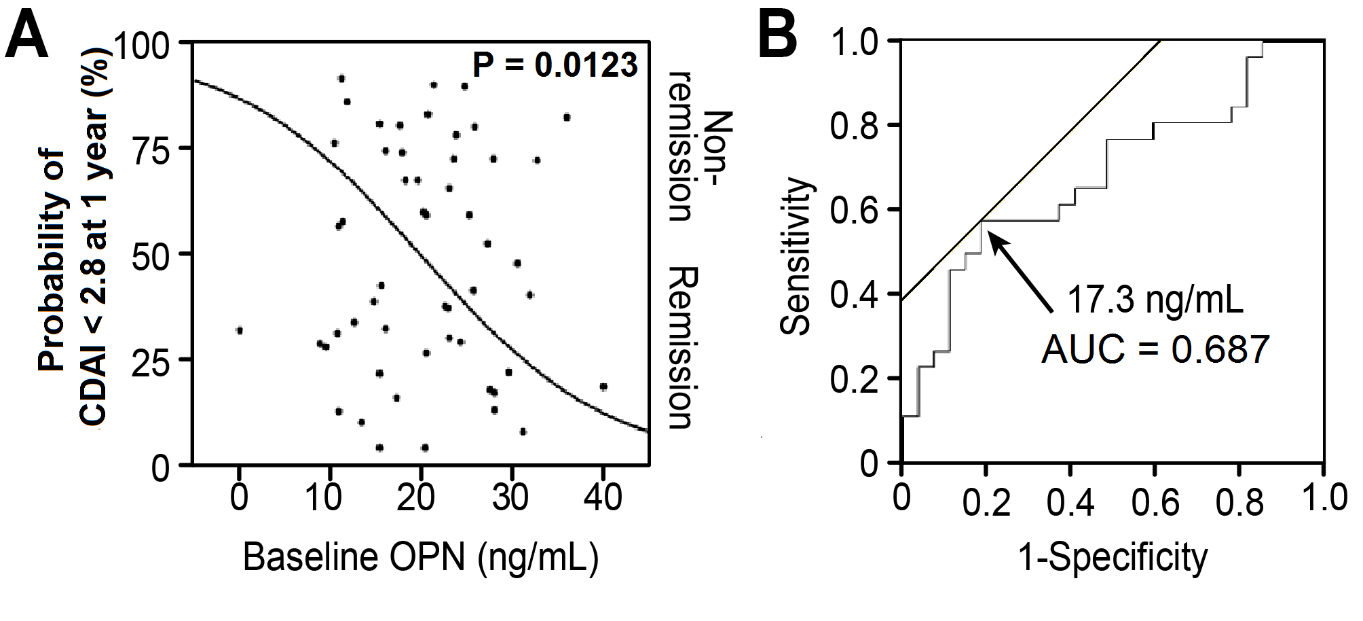

Supplement: S5 Fig — (TIF) [file pone.0152341.s001.tif]
